# Clinical usefulness of measurement of plasma soluble fibrin levels in critically ill patients

**DOI:** 10.1186/cc13397

**Published:** 2014-03-17

**Authors:** T Masuda, T Kobayashi, H Takahashi, K Yoshikawa,, M Takahashi, E Isotani

**Affiliations:** 1 Tokyo Women's Medical University Medical Center East, Tokyo, Japan

## Introduction

The soluble fibrin monomer fibrinogen complex (SF) is a complex coupling fibrin monomer and fibrinogen molecules. As the level of SF reflects the thrombin generation activity in plasma, we may estimate the early-activated state of blood coagulation by the measurement of SF. The aim of this study is to evaluate the clinical usefulness of SF for the hypercoagulated state.

## Methods

We measured the plasma level of SF in 63 patients within 48 hours after admission and on the 1st, 3rd, 5th and 7th days after admission. Underlying disease mainly includes sepsis, shock, and so on. According to the disseminated intravascular coagulation diagnostic criteria established by the Japanese Association of Acute Medicine, we defined the DIC group as JAAM-DIC score more than 3 within 48 hours after admission, the Subclinical DIC group as score more than 3 within 7 days beyond 48 hours after admission, and the No DIC group as score less than 4 during the entire study period. The SF value of each group was compared with the Mann-Whitney U test.

## Results

**Figure 1:**
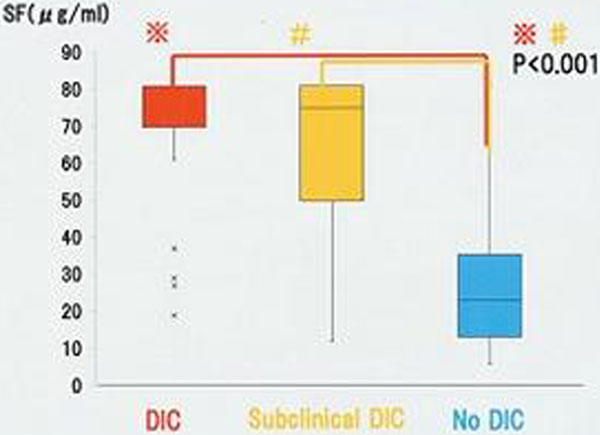
**SF values of the DIC, Subclinical DIC and No DIC groups**.

**Figure 2:**
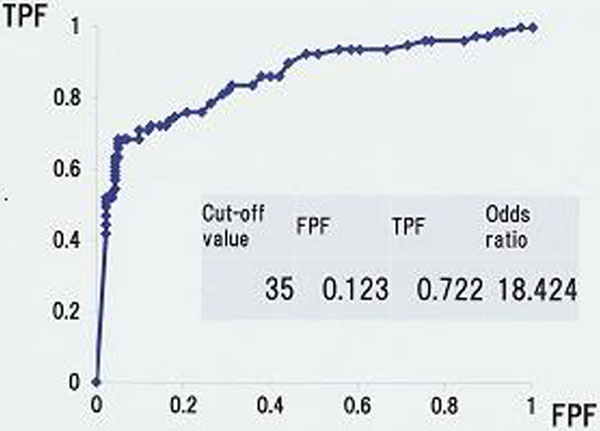
**ROC curve of the SF value for DIC**.

The SF values in the DIC and the Subclinical DIC groups were significantly higher than in the No DIC group. We created the receiver operating characteristic curve of SF value for DIC onset (JAAM-DIC score ≥4) and the SF value of 35 μg/ml was set as the cutoff SF value. The high SF group (SF ≥35 μg/ml) had significantly higher JAAM-DIC score, SOFA score and APACHE II score than the low SF group (SF <35 μg/ml). Mainly in the high SF group except DIC patients on admission, we found that SF increased before the JAAM-DIC score changed. See Figures 1 and 2.

## Conclusion

We think measurement of the plasma SF level may be clinically useful in evaluating the severity of critically ill patients such as those with sepsis.

